# Structural MRI study of Pareidolia and Visual Hallucinations in Drug–Naïve Parkinson’s disease

**DOI:** 10.1038/s41598-024-82707-x

**Published:** 2024-12-28

**Authors:** Masakazu Ozawa, Tomotaka Shiraishi, Hidetomo Murakami, Daisuke Yoshimaru, Asako Onda, Hiromasa Matsuno, Teppei Komatsu, Kenichi Sakuta, Kenichiro Sakai, Tadashi Umehara, Hidetaka Mitsumura, Hirotaka James Okano, Yasuyuki Iguchi

**Affiliations:** 1 Department of Neurology, The Jikei University School of Medicine, 3-25-8 Nishi-Shimbashi, Minato-ku, Tokyo, 105-8461 Japan; 2 Division of Regenerative Medicine, The Jikei University School of Medicine, Tokyo, Japan; 3 Department of Neurology, Showa University School of Medicine, Tokyo, Japan

**Keywords:** Pareidolia, Visual hallucination, Minor hallucination, Parkinson’s disease, Surface-based morphometry, Parkinson's disease, Parkinson's disease

## Abstract

Visual hallucinations (VH) and pareidolia, a type of minor hallucination, share common underlying mechanisms. However, the similarities and differences in their brain regions remain poorly understood in Parkinson’s disease (PD). A total of 104 drug-naïve PD patients underwent structural MRI and were assessed for pareidolia using the Noise Pareidolia Test (NPT) were enrolled. Subcortical gray matter volume and cortical surface volume were analyzed using the FreeSurfer software. Structural analyses revealed associations between NPT scores and atrophy in the right thalamus, right hippocampus, right temporal cortex, and right orbitofrontal cortex in all PD participants. These results were almost the same after adjusting for right-handed 97 patients with PD. It is considered that hallucinations in patients with PD are related to altered integration of sensory input (bottom-up) and prior knowledge (top-down) within the visual system. Our findings indicate that pareidolia in PD involves both bottom-up (thalamus and temporal cortex) and top-down (orbitofrontal cortex) processing disturbances; in contrast, VH predominantly involves bottom-up but not top-down regions. Understanding these distinctions could aid in the development of targeted interventions for hallucinations in patients with PD.

## Introduction

Psychiatric symptoms are common in Lewy body diseases (LBD), such as Parkinson’s disease (PD) and dementia with Lewy bodies (DLB). These symptoms significantly impact caregiver burden^1^, decrease quality of life^2^, and increase mortality rates^3^. Psychiatric symptoms associated with LBD include visual hallucinations (VH), minor hallucinations (MH), and delusions. MH includes pareidolia (misperceptions of real external stimuli), sense of presence (a vivid sensation that someone is present nearby), and passage hallucinations (observation of an animal or object passing through the peripheral visual field)^4^. One previous longitudinal study involving 250 patients with PD reported that the prevalence of VH was 7%, while that of MH was 20% (including 6% for pareidolia, 3.6% for sense of presence, and 18% for passage hallucinations), with nearly 40% of those experiencing MH also having concomitant VH^5^. MH often precedes the onset of VH and is associated with rapid eye movement sleep behavior disorder (RBD) and depression in patients with PD^6^. Although the pathogenesis of MH and VH in PD are similar, MH does not always appear first, and is considered a distinct conditions^7^. Among these symptoms, VH and pareidolia have drawn attention due to their overlapping yet distinct mechanisms.

MH is linked to the loss of gray matter volume (GMV) in visuoperceptive areas and subjective cognitive decline in drug-naïve PD, as measured using the hallucination questionnaire in the Movement Disorder Society-Unified Parkinson’s Disease Rating Scale (MDS-UPDRS) part I^8^. Bejr-Kasem et al. compared GMV in PD patients with MH (PD-MH) and without MH (PD-noMH), using a hallucination questionnaire in MDS-UPDRS part I. GMV in PD-MH was reduced in the left posterior cingulate cortex, right fusiform, right parahippocampal, right precuneus, left middle-occipital, left supramarginal, and left angular cortices compared to PD-noMH^9^. The affected functional brain connectivity associated with PD-MH involves visual processing areas and attention control networks, which overlap with the abnormalities described in PD patients with VH (PD-VH)^10^. Using ^18^F-fluorodeoxyglucose-positron emission tomography imaging, Nishio et al. revealed that the regional glucose metabolic rate in the posterior cingulate and medial occipitoparietal cortices was lower in PD-MH than PD-noMH^11^. Previous reports have identified regions of brain atrophy in both PD-MH and PD-VH, with some overlapping areas, but also many differences.

Pareidolia, which is complex visual illusions involving ambiguous forms that are perceived as meaningful objects, has recently gained attention. The 40-item noise pareidolia test (NPT) was established to differentiate DLB from Alzheimer’s disease and normal population (81% sensitivity and 92% specificity)^12^. The 20‐item version NPT, which was selected from the 40-item NPT, is useful in detecting pareidolia in patients with PD (cut-off of 1 yielded 75% sensitivity and 81% specificity)^13^. Kurumada et al. previously showed that the positivity rate of NPT in patients with PD without VH was 47.5% and that of multiple system atrophy (MSA) was 18.8%, suggesting it may be a highly specific test for differentiating PD from MSA^14^. Pareidolia reflects visual illusions, but may also reflect susceptibility to VH in patients with LBD^15^.

Previous reports have bundled three symptoms of MH (pareidolia, sense of presence, and passage hallucinations), making it difficult to compare MH with VH. Although PD-MH, including pareidolia, and PD-VH share common underlying mechanisms, the similarities and differences in their brain regions remain poorly understood. A few previous reports evaluating structural MRI data of PD-MH selected participants through interviews or MDS-UPDRS part I, but did not use the NPT, which was established to evaluate pareidolia in PD. Unlike few previous studies that used general questionnaires such as MDS-UPDRS part I, which often conflate VH and MH, this study employed the NPT—a specific and validated tool for assessing pareidolia and its severity. This approach allows for a more precise evaluation, reducing the potential for overlapping symptoms. Additionally, because anti-parkinsonian drugs can induce VH and MH, focusing on only drug-naïve PD patients is essential to understand the pathology of hallucinations^16^. Therefore, in the present study, we assessed surface-based morphometry (SBM) in drug-naïve PD patients selected using established NPT scores and a history of VH to compare the regions between pareidolia and the VH. This study contributes to a better understanding of the pathophysiological mechanisms underlying pareidolia in patients with PD.

## Results

### Participant characteristics

A total of 104 patients with PD (63.5% male, 93.3% right-handed dominant) were enrolled in this study. Among them, 35 (33.7%) had pareidolia and 8 (7.7%) had VH (Table 1). The NPT score significantly correlated with age (r = 0.283, p = 0.002), MDS-UPDRS part III score (r = 0.191, p = 0.027), FAB score (r = − 0.192, p = 0.026), RBDSQ score (r = 0.298, p = 0.001), and VH (r = 0.372, p < 0.001).

**Table 1:** Clinical profiles and symptoms, and Pearson’s correlation coefficients of noise pareidolia test.

	All participants	Pearson’s correlation test (vs noise Pareidolia test)	
	n = 104	r	p value
Age (years)	68.9 ± 9.7 (43–85)	0.283	0.002**
Male, n (%)	66 (63.5%)	0.067	0.249
right dominant hand, n (%)	97 (93.3%)	0.006	0.474
Disease duration (years)	2.3 ± 2.1 (0–11)	0.027	0.391
MDS-UPDRS part III	26.7 ± 13.6 (4–83)	0.191	0.027*
MMSE	27.9 ± 2.8 (18–30)	− 0.192	0.026*
FAB	15.9 ± 2.5 (6–18)	− 0.242	0.007**
RBDSQ	2.8 ± 2.6 (0–13)	0.298	0.001**
Visual hallucination positive, N (%)	8 (7.7%)	0.372	< 0.001**
Noise pareidolia test	2.4 ± 5.8 (0–34)		
Noise pareidolia test positive, n (%)	35 (33.7%)		

*MDS-UPDRS* Movement Disorder Society-Unified Parkinson’s Disease Rating Scale, *MMSE* Mini–Mental State Examination, *FAB* frontal assessment battery, *RBDSQ* rapid eye movement sleep behaviour disorder screening questionnaire.

*p < 0.05, **p < 0.01.

### Volume loss of subcortical gray matter by Pareidolia or Visual Hallucination

In the multiple regression analyses on the subcortical GMV, the NPT score was found to predict the reduced volume of the right thalamus (β = − 0.204, p = 0.001) and right hippocampus (β = − 0.198, p = 0.006) after correcting for FDR < 0.05 (Table 2). In the same way, VH predicted the right thalamus (β = − 0.190, p = 0.002), right putamen (β = − 0.236, p = 0.004), and right hippocampus (β = − 0.184, p = 0.007) (Table 3). Furthermore, in the analyses of the 97 right-handed PD patients, the NPT score predicted the right thalamus (β = − 0.211, p = 0.001) (Table S1), while VH predicted the right thalamus (β = − 0.218, p < 0.001), right putamen (β = − 0.235, p = 0.005), and right hippocampus (β = − 0.219, p = 0.002) (Table S2).

**Table 2:** Multiple regression models for Pareidolia on subcortical gray matter volume.

		Age		Sex		TIV		Noise Pareidolia test				
		β	p value	β	p value	β	p value	B	95% CI	β	p value	q value
Thalamus	Left	− 0.427	< 0.001	− 0.123	0.079	0.727	< 0.001	9.478	− 8.1 to 27.0	0.064	0.286	0.400
	Right	− 0.332	< 0.001	− 0.007	0.926	0.642	< 0.001	− 35.795	− 57.1 to − 14.5	** − 0.204**	0.001*	**0.014**
Caudate	Left	− 0.217	0.013	− 0.045	0.651	0.509	< 0.001	− 8.854	− 24.4 to 6.6	− 0.098	0.260	0.400
	Right	− 0.151	0.077	− 0.035	0.723	0.508	< 0.001	− 21.592	− 38.9 to − 4.2	− 0.210	0.015	0.053
Putamen	Left	− 0.409	< 0.001	0.096	0.304	0.404	< 0.001	5.574	− 12.1 to 23.2	0.051	0.532	0.667
	Right	− 0.297	< 0.001	0.072	0.459	0.369	< 0.001	− 26.105	− 46.8 to − 5.4	− 0.209	0.014	0.053
Pallidum	Left	− 0.212	0.027	0.009	0.938	0.367	0.001	0.611	− 7.2 to 8.4	0.015	0.877	0.889
	Right	− 0.015	0.873	− 0.085	0.434	0.417	< 0.001	− 10.558	− 19.4 to − 1.7	− 0.223	0.020	0.056
Hippocampus	Left	− 0.439	< 0.001	0.002	0.978	0.537	< 0.001	0.843	− 11.1 to 12.8	0.010	0.889	0.889
	Right	− 0.443	< 0.001	− 0.063	0.436	0.495	< 0.001	− 17.760	− 30.2 to − 5.3	** − 0.198**	0.006*	**0.042**
Amygdala	Left	− 0.384	< 0.001	0.051	0.619	0.279	0.007	− 4.228	− 11.7 to 3.2	− 0.099	0.261	0.400
	Right	− 0.365	< 0.001	0.206	0.030	0.269	0.005	− 7.129	− 13.7 to − 0.6	− 0.174	0.034	0.079
Accumbens	Left	− 0.495	< 0.001	0.001	0.991	0.353	< 0.001	− 0.552	− 3.5 to 2.5	− 0.028	0.730	0.852
	Right	− 0.471	< 0.001	− 0.049	0.607	0.315	0.001	− 2.201	− 5.3 to 0.9	− 0.118	0.156	0.312

*TIV* total intracranial volume, *95% CI* 95% confidence interval.

Significant values are given in bold.

*Represents p < 0.05 after FDR correction.

**Table 3:** Multiple regression models for visual hallucination on subcortical gray matter volume.

		Age		Sex		TIV		Visual hallucination				
		β	p value	β	p value	β	p value	B	95% CI	β	p value	q value
Thalamus	Left	− 0.414	< 0.001	− 0.108	0.123	0.715	< 0.001	− 152.037	− 518.6 to 214.5	− 0.048	0.412	0.444
	Right	− 0.409	< 0.001	− 0.014	0.848	0.644	< 0.001	− 723.296	− 1168.4 to − 278.2	− **0.190**	0.002*	**0.028**
Caudate	Left	− 0.258	0.002	− 0.045	0.652	0.507	< 0.001	− 243.241	− 564.9 to 78.5	− 0.124	0.137	0.202
	Right	− 0.227	0.007	− 0.046	0.641	0.513	< 0.001	− 360.142	− 725.9 to 5.7	− 0.161	0.054	0.116
Putamen	Left	− 0.403	< 0.001	0.113	0.227	0.391	< 0.001	− 177.922	− 545.0 to 189.2	− 0.074	0.339	0.396
	Right	− 0.380	< 0.001	0.069	0.467	0.367	< 0.001	− 638.764	− 1064.9 to − 212.6	− **0.236**	0.004*	**0.028**
Pallidum	Left	− 0.215	0.020	0.019	0.864	0.359	0.001	− 60.065	− 222.8 to 102.7	− 0.066	0.466	0.466
	Right	− 0.096	0.298	− 0.097	0.377	0.423	< 0.001	− 177.828	− 363.9 to 8.3	− 0.173	0.061	0.116
Hippocampus	Left	− 0.445	< 0.001	0.015	0.860	0.526	< 0.001	− 162.727	− 410.7 to 85.3	− 0.089	0.196	0.249
	Right	− 0.517	< 0.001	− 0.070	0.389	0.497	< 0.001	− 359.392	− 620.1 to − 98.7	− **0.184**	0.007*	**0.033**
Amygdala	Left	− 0.429	< 0.001	0.056	0.580	0.273	0.008	− 148.567	− 301.6 to 4.5	− 0.161	0.057	0.116
	Right	− 0.429	< 0.001	0.198	0.038	0.273	0.005	− 129.06	− 267.1 to 8.9	− 0.146	0.066	0.116
Accumbens	Left	− 0.515	< 0.001	0.011	0.909	0.344	< 0.001	− 45.858	− 107.6 to 15.9	− 0.113	0.144	0.202
	Right	− 0.521	< 0.001	− 0.047	0.621	0.310	0.001	− 66.582	− 129.5 to − 3.6	− 0.165	0.038	0.116

*TIV* total intracranial volume.

Significant values are given in bold.

*Represents p < 0.05 after FDR correction.

### Cortical volume correlated with Pareidolia or Visual Hallucination

**Fig. 1:**
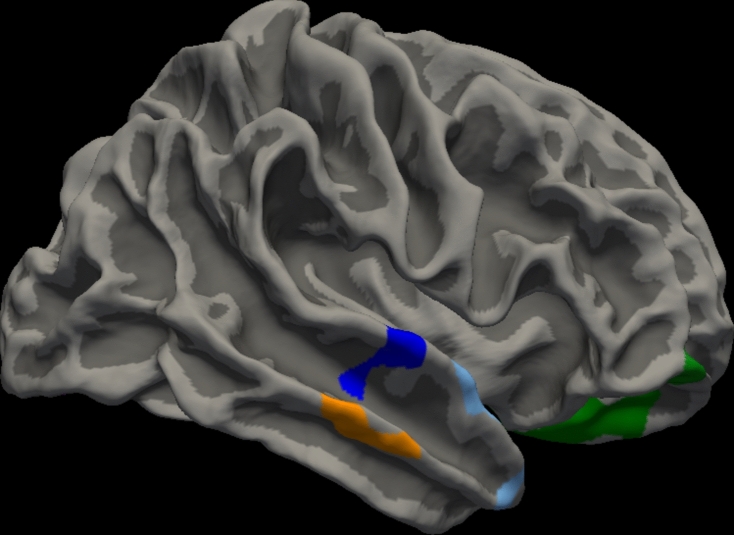
Surface-based morphometry analysis revealed that the score of Noise Pareidolia Test was associated with cortical volume in right medial orbitofrontal cortex (green), right lateral orbitofrontal cortex (green), right superior temporal cortex (blue), right middle temporal cortex (orange), right temporal pole (light blue), and right lateral occipital cortex (pink). (p < 0.01 corrected by Monte Carlo simulation).

**Fig. 2:**
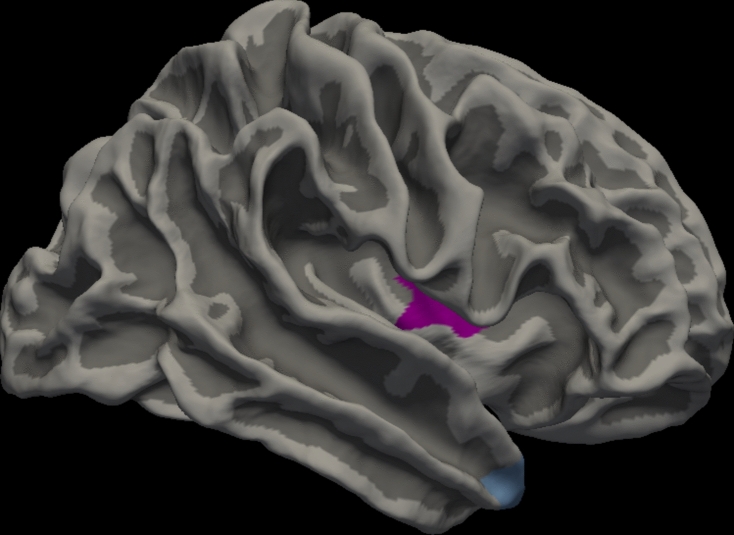
Surface-based morphometry analysis revealed that the Visual Hallucination was associated with cortical volume in right insula (purple) and right temporal pole (light blue) (p < 0.01 corrected by Monte Carlo simulation).

The NPT score was significantly associated with the surface volumes of the right medial orbitofrontal cortex, right lateral orbitofrontal cortex, right superior temporal cortex, right middle temporal cortex, right temporal pole, and right lateral occipital cortex (p < 0.01, corrected using Monte Carlo simulation) (Fig. 1, S1). Similarly, VH was found to be significantly associated with the right insula and right temporal pole (Fig. 2). Furthermore, in the analysis of 97 right-handed dominant patients with PD, the NPT score was significantly associated with the surface volume of the right medial orbitofrontal cortex, right lateral orbitofrontal cortex, right middle temporal cortex, and right superior temporal cortex (Fig. S2). In these right-handed-dominant PD patients, VH was significantly associated with the right insula and right temporal pole (Fig. S3).

## Discussion

In this study, we performed detailed clinical and imaging analyses to identify regions associated with pareidolia or VH in drug-naïve patients with PD. Pareidolia and VH are thought to share common brain regions and mechanisms; however, the detailed differences between them are only poorly understood. Our findings identified the right thalamus, right hippocampus, and right temporal pole as common atrophic regions in both pareidolia and VH. However, the right orbitofrontal cortex, right temporal cortex, and right lateral occipital cortex were associated with pareidolia, whereas the right putamen and right insula were associated with VH. These results indicate that while pareidolia and VH share some common regions, they also exhibit distinct pathological features. Considering our results, we proposed atrophy of the right orbitofrontal, right temporal, and right lateral occipital cortices as potential mechanisms underlying pareidolia, distinct from VH, in patients with PD. Because only the right hemisphere showed significant differences in this study, the results were almost the same after adjusting for only right-handed patients with PD. Our findings are consistent with the well-established lateralization of visuospatial attention in the right hemisphere and language production in the left hemisphere^17^. The atrophy of the hippocampus and occipital cortex was consistent with the results of previous MH studies. However, our observation of atrophy of the thalamus, insula, and orbitofrontal cortex is inconsistent. Previous neuroimaging studies on the GMV in patients with PD have shown inconsistent results regarding hallucinations^18^. These differences may be due to heterogeneity in age, sex, disease duration, and disease severity among patients with PD.

Visual information is processed via the ventral and dorsal pathway^19^. The dorsal visual pathway, also called the “where pathway,” is an occipito-parietal network of cortical areas that supports spatial cognition and the real-time transformation of visual input into motoric output^20^. The ventral visual pathway, also termed the “what pathway,” is an occipito-temporal network that supports the recognition of object identity, including the visual perception of faces, by bridging visual input with information stored in long-term memory, such as semantic and episodic knowledge^21^. This network extends from the occipital lobe to the ventral and lateral regions of the temporal lobe^22^. When humans recognize objects, information is input into the brain through bottom-up processes, and subsequently perceived through top-down processes. Visual information that enters the retina passes through the optic nerve and the lateral geniculate nucleus (part of the thalamus) before being projected onto the primary visual cortex (occipital cortex)^23^. Thereafter, it splits into the ventral visual pathway, which leads to the temporal lobe, and the dorsal visual pathway, which leads to the parietal lobe. The input of information from the periphery to the central nervous system is known as bottom-up processing. Next, information input through bottom-up processing is modified in the frontal lobe, taking into account memories (hippocampus) and emotions (amygdala)^24^. The recognition of this input information by the frontal lobe, including the orbitofrontal cortex, is referred to as top-down processing^25^. In patients with PD, VH is related to the altered integration of sensory input (bottom-up) and prior knowledge (top-down) within the visual system^26^. Several studies have previously reported functional connectivity reduction between bottom-up processing and the right insula, right hippocampus, left putamen, and bilateral caudate in PD-VH compared to healthy controls^27,28^.

In our results, the VH showed atrophy in regions associated with bottom-up processing (right thalamus, right insula, right putamen, and right temporal pole) and the right hippocampus. Pareidolia exhibited atrophy not only in the bottom-up regions consistent with VH, but also in regions associated with top-down processing (right orbitofrontal cortex). These findings indicate that while both pareidolia and the VH are influenced by bottom-up processing disturbances, pareidolia are more influenced by top-down processing disturbances than the VH. While VH is characterized by seeing things that are not present, pareidolia misinterprets actual objects, indicating that top-down disturbances play a significant role in the mechanisms underlying pareidolia. The orbitofrontal cortex lies at the interface of emotion and cognition, and tends to be activated by stimuli such as rewards or punishments^29^. The hippocampal to medial orbitofrontal cortex projections are conditionally necessary for value updating, and are involved in long-term aversion-based value memory updating^30^. We considered that patients with PD suffer from pareidolia due to atrophy of the bottom-up regions (right thalamus, right temporal cortex, and right lateral-occipital cortex), right hippocampus, and top-down regions (right medial-orbitofrontal cortex and right lateral-orbitofrontal cortex). Previous studies support our findings. Kravitz et al. reported that the dorsal visual pathway to the middle temporal cortex, involving the hippocampus, is important for visuospatial cognition according to memory^31^. Nagy et al. further reported that the lateral occipital cortex plays an important role in the perception of facial stimuli^32^. In addition, Pehrs et al. reported that the temporal pole controls lower-level perceptual areas in the ventral visual stream, such as the superior temporal and fusiform cortices, and acts as a domain-general hub integrating socioemotional information^33^.

Pathological reports of LBD have suggested that limbic Lewy body pathology is associated with an earlier onset of VH, whereas cortical Lewy body pathology is not. However, both limbic and cortical Lewy body pathologies are associated with visual misperception and misidentification^34^. Harding et al. previously reported that VH in LBD are associated with a greater Lewy body burden in the temporal lobe^35^. Gallagher et al. further suggested that the PD-VH had significantly higher Lewy body densities in the middle frontal, middle temporal, transentorhinal, and anterior cingulate cortices^36^. Based on these pathological findings, Lewy bodies in the temporal and orbitofrontal cortices can induce pareidolia and VH. Previous functional MRI study on DLB patients has shown a reduced response to the visual stimuli in the middle temporal lobe^37^. These findings support that visual association areas, such as the temporal lobe, may contribute to VH in patients with LBD.

In our study, the NPT score was found to be strongly associated with age, FAB score, RBDSQ score, and VH and slightly associated with the MDS-UPDRS part III and MMSE scores. Therefore, the NPT score was significantly associated with a decline in cognitive function that could be detected using a cognitive assessment battery specifically used to assess frontal function (FAB score) rather than general cognitive assessments (MMSE score). As such, our study revealed that both structural orbitofrontal atrophy and functional frontal cognitive decline were correlated with pareidolia in patients with PD. RBD has been previously demonstrated to be significantly associated with MH^6^. Similarly, our study revealed that pareidolia was significantly associated with RBDSQ score.

This study had several limitations. First, we evaluated pareidolia using the NPT; however, we did not assess passage hallucinations or the sense of presence. Furthermore, the evaluation of VH relied on clinical examination, without the use of sensitive or standardized assessment tools, which may have limited the accuracy and robustness of our findings. As such, further tests utilizing machines or questionnaires should be conducted to evaluate passage hallucinations, sense of presence, and VH more accurately. Second, we were unable to recruit control participants, which limits our ability to determine whether the atrophic regions associated with pareidolia and VH are specific to patients with PD or also occur in healthy individuals. To address this gap, future studies should incorporate healthy control participants to better differentiate between PD-specific atrophic changes and normal age-related brain variations. This approach would help elucidate the pathophysiology of hallucinations in PD and clarify whether these structural changes are unique to the disease or part of a broader spectrum of age-related neural alterations. Third, almost all our participants were right-handed individuals with PD, resulting in an insufficient number of left-handed participants to conduct meaningful statistical analyses. By recruiting more left-handed patients with PD, we may be able to better understand if the lateralization of neural changes observed in this study is consistent across different handedness groups or if variations exist. This could provide deeper insights into the role of hemispheric dominance in the development of visual disturbances in PD. Fourth, our analyses of cortical and subcortical gray matter volume included age, sex, and TIV as covariates; however, education years, disease severity, and cognitive status were not accounted for. Including additional covariates such as detailed motor and non-motor severity measures could enhance the precision of identifying brain regions associated with pareidolia and VH by reducing potential confounding effects. Fifth, while we identified atrophic regions associated with pareidolia and VH, we did not investigate the connectivity between these regions. Further studies using functional MRI or structural connectivity analyses will be essential to confirm whether these regions are linked via disrupted top-down or bottom-up pathways. Sixth, due to our focus on recruiting drug-naïve patients in the early stages of PD, the prevalence of pareidolia and VH in our cohort was very low. This led to small sample sizes for subgroup analyses, resulting in statistical limitations that may affect the robustness and generalizability of our findings. Direct comparisons of cortical structural differences between pareidolia and VH were not conducted due to the small sample size of VH-positive patients. Future studies with larger sample sizes and broader patient populations are necessary to validate these results.

In conclusion, atrophy of the right orbitofrontal cortex, right hippocampus, right thalamus, and right temporal cortex is associated with the severity of pareidolia in drug-naïve patients with PD. The atrophic regions of pareidolia and VH share some commonalities and differences, suggesting that they are similar but distinct pathologies. These distinctions could serve as potential biomarkers for the early diagnosis and therapeutic targets for hallucinations in patients with PD.

## Methods

### Participants

This was a retrospective cohort study of patients with PD who were admitted to the Jikei University Hospital between September 2016 and March 2024. This study was approved by the Ethics Committee of the Jikei University School of Medicine (approval number 27-315 [8200]), and informed consent was obtained from all participants. This study was conducted in accordance with the principles of the Declaration of Helsinki. We reviewed the medical records of 174 consecutive PD patients who had never received anti-parkinsonian drug therapy. The exclusion criteria for this cohort included (Fig. S4): (1) DLB participants with dementia developing before or within 1 year after the onset of parkinsonism (n = 9), (2) participants who were not diagnosed with “clinically established” or “probable” PD by the Movement Disorder Society (MDS) Clinical Diagnostic Criteria for Parkinson’s disease^38^ (n = 6), (3) participants who did not undergo MRI in a scanner at the Jikei University Hospital (n = 48), and (4) participants who did not undergo the NPT (n = 7). Consequently, 104 participants were enrolled in this study. In the clinical assessments, age, sex, dominant hand, and disease duration were evaluated in all participants. Motor symptoms were assessed using the MDS-UPDRS Part III. The Mini–Mental State Examination (MMSE)^39^ and the Frontal Assessment Battery (FAB)^40^ were used to measure the cognitive status. The RBD was assessed using the RBD screening questionnaire (RBDSQ)^41^. The presence of VH was assessed through structured clinical interviews conducted by neurology specialists. During these interviews, patients were carefully questioned about whether they had experienced seeing people, children, animals, insects, or other objects in places where nothing was present.

### Noise Pareidolia test

The symptom of pareidolia was assessed using the NPT, which was developed at Tohoku University^12,42^. The test comprised 40 black and white visual noise images with dimensions of 16 cm × 16 cm. Among these images, a person’s face was found to be embedded in eight images. During the assessment, the participants were presented with all 40 images, and asked to indicate whether they perceived a face in each image. Each image was displayed for a maximum of 60 s, and the participants received no feedback, regardless of the accuracy of their responses. When subjects responded with comments such as “It looks like a face,” we asked the subjects whether there was an actual face in the noise stimuli, or whether the subject saw something that simply looked like a face. Only the former response was considered paradolic. If a participant reported noticing an illusory face in at least one image without an embedded face, pareidolia was deemed to be present. The degree of pareidolia was quantified by the number of images in which the participants falsely perceived an illusory face. The degree of pareidolic response is suitable for assessing the severity of visual illusion (pareidolia) and was used as a continuous variable in this study.

### MRI acquisition and preprocessing for FreeSurfer analysis

All participants underwent MRI using a 3 T system (Siemens Healthcare, Erlangen, Germany), equipped with a 20-channel head–neck coil. Structural 3D T1-weighted images were acquired using a magnetization-prepared rapid gradient-echo (MPRAGE) pulse sequence (repetition time, 1800 ms; echo time, 2.85 ms; flip angle, 10°; field of view, 240 mm; matrix, 256 × 256; voxel size, 0.94 × 0.94 mm; thickness, 1.0 mm; acquisition time, 283 s). The 3D T1-weighted images were processed using the FreeSurfer pipeline version, 7.3.2 (http://surfer.net/) to calculate the cortical volume using surface-based analysis^43,44^. Each cortical segmentation was visually inspected for inaccuracies, and manually corrected using correction tools provided by FreeSurfer. Additionally, automatic subcortical segmentation was performed using FreeSurfer^45^, while the volumes of the bilateral thalamus, amygdala, hippocampus, globus pallidus, caudate nucleus, putamen, and accumbens nucleus were calculated. Moreover, the total intracranial volume (TIV) was included as a covariate in the analysis to adjust for interpatient differences in head size. We utilized the Desikan-Killiany atlas, which parcellates the cortical surface into 34 regions per hemisphere.

### Statistical analysis

All analyses were performed using IBM SPSS Statistics (version 29.0; IBM Corporation, Armonk, NY, USA), except for Benjamini–Hochberg adjustments, which were performed using Microsoft Excel (version 16.81; Redmond, WA, US). Continuous variables are presented as the means ± standard deviations, while categorical variables are presented as frequencies (percentages). Statistical significance was set at p < 0.05. Pearson’s correlation test was applied to examine the relationship between the NPT scores and background variables. To accurately assess the impact of pareidolia on the subcortical gray matter, multiple regression analyses were conducted using each subcortical GMV as a dependent variable and age, sex, TIV, and NPT score as independent variables. Similarly, to assess the impact of VH, multiple regression analyses were performed with age, sex, TIV, and presence of VH as independent variables. To limit the false discovery rate (FDR), Benjamini–Hochberg adjusted p-values were calculated with a significance threshold set at p < 0.05. For the SBM analyses of surface volume, we examined the main and interaction effects of NPT and VH after adjusting for age and sex. Corrections for multiple comparisons were conducted using a Monte Carlo simulation (10,000 iterations) with a cluster-wise threshold of p < 0.01 in SBM. To minimize the influence of differences between the dominant and inferior hemispheres, 97 right-handed PD patients were selected for further analyses.

## Supplementary Information

Supplementary Information 1.

Supplementary Information 2.

Supplementary Information 3.

Supplementary Information 4.

Supplementary Information 5.

Supplementary Information 6.

## Data Availability

The datasets used and/or analysed during the current study available from the corresponding author on reasonable request.
