# Supplementary material for: Structural MRI study of Pareidolia and Visual Hallucinations in Drug–Naïve Parkinson’s disease
Source: Sci Rep. 2024 Dec 28;14:31293. doi: 10.1038/s41598-024-82707-x (PMC11682137; doi:10.1038/s41598-024-82707-x)
Supplement: Supplementary file 1 — Supplementary Information 1. [file 41598_2024_82707_MOESM1_ESM.pptx]

## Slide 1
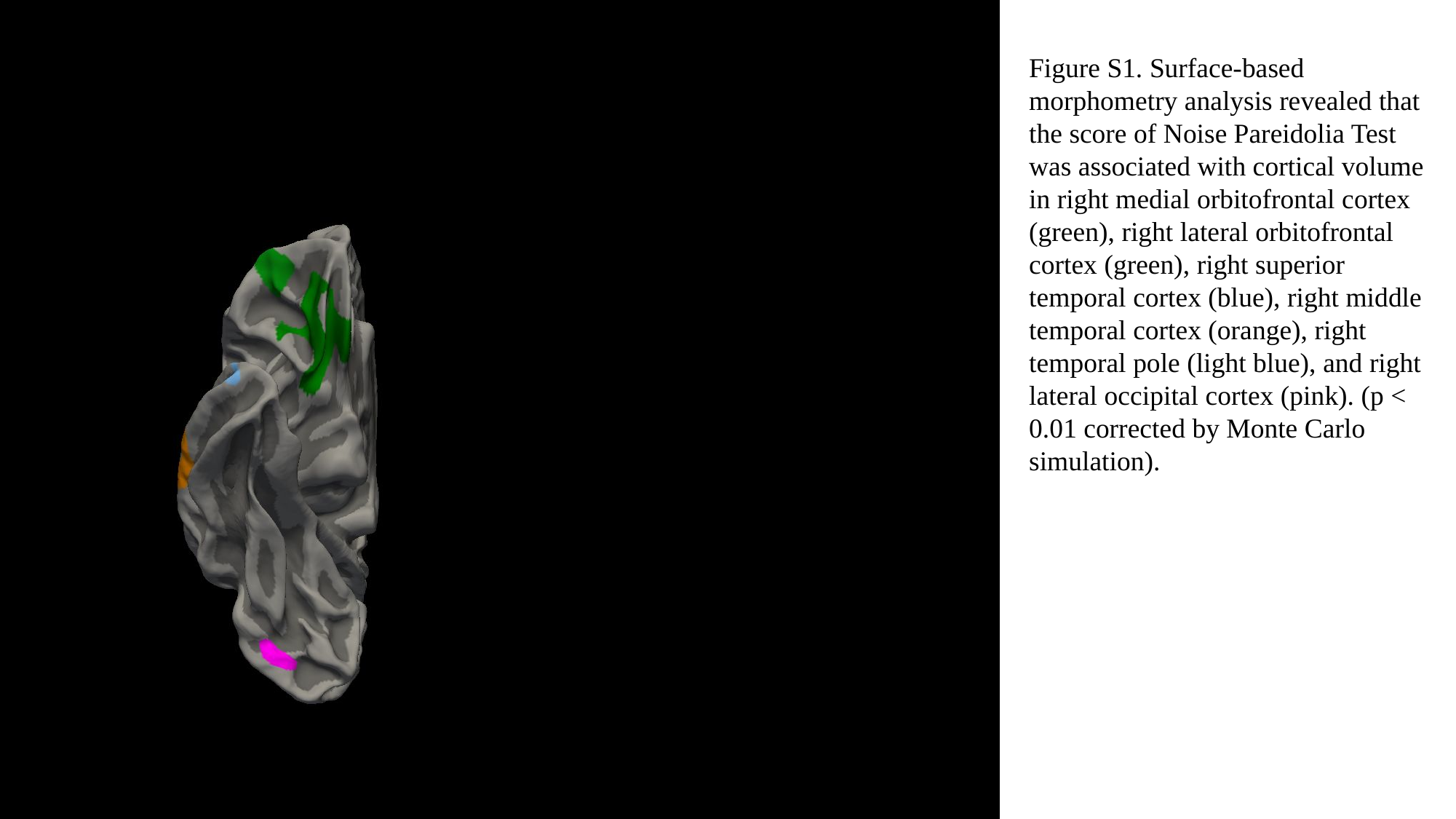

Figure S1. Surface-based morphometry analysis revealed that the score of Noise Pareidolia Test was associated with cortical volume in right medial orbitofrontal cortex (green), right lateral orbitofrontal cortex (green), right superior temporal cortex (blue), right middle temporal cortex (orange), right temporal pole (light blue), and right lateral occipital cortex (pink). (p < 0.01 corrected by Monte Carlo simulation).
