# Supplementary material for: Structural MRI study of Pareidolia and Visual Hallucinations in Drug–Naïve Parkinson’s disease
Source: Sci Rep. 2024 Dec 28;14:31293. doi: 10.1038/s41598-024-82707-x (PMC11682137; doi:10.1038/s41598-024-82707-x)
Supplement: Supplementary file 2 — Supplementary Information 2. [file 41598_2024_82707_MOESM2_ESM.pptx]

## Slide 1
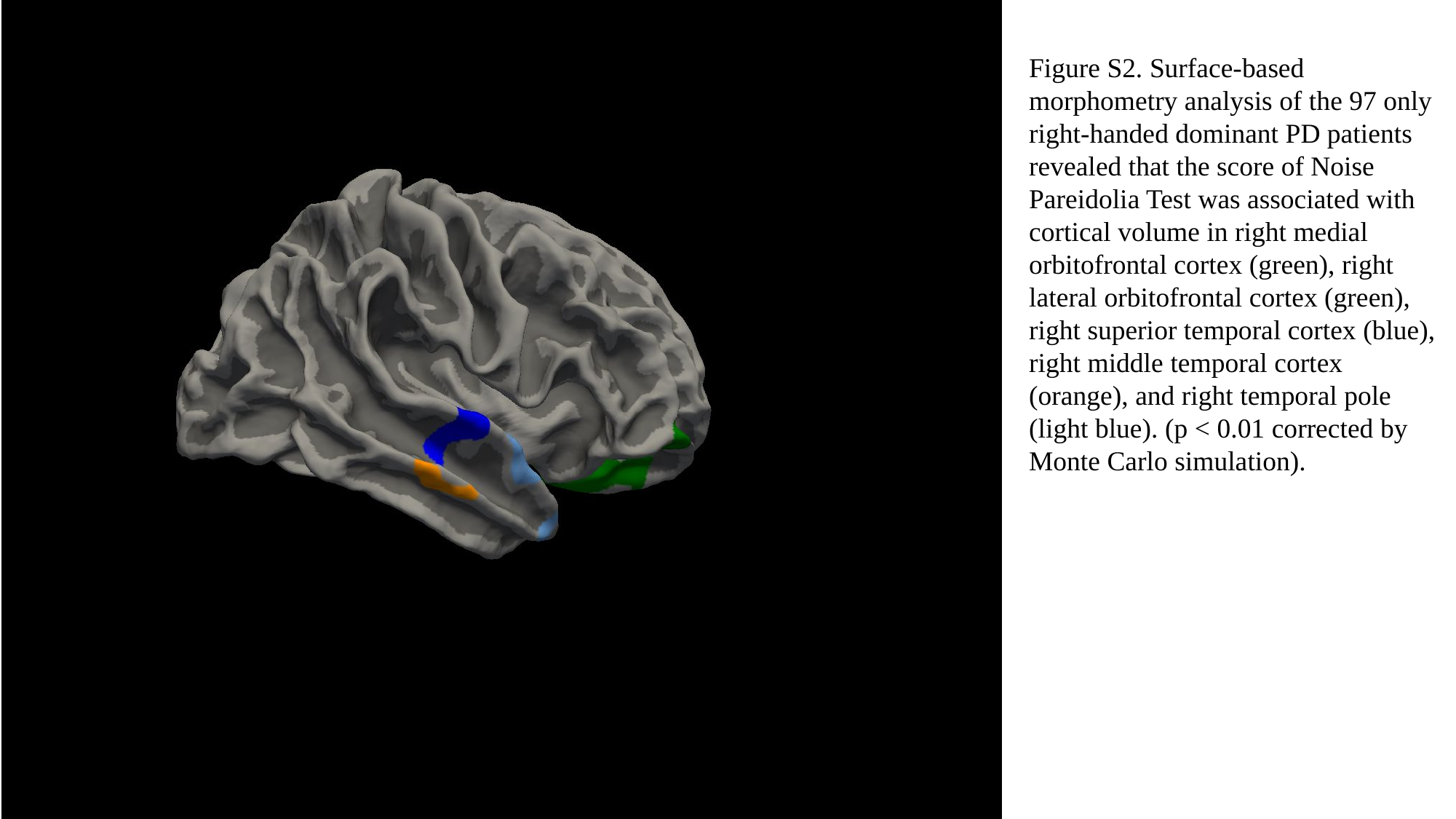

Figure S2. Surface-based morphometry analysis of the 97 only right-handed dominant PD patients revealed that the score of Noise Pareidolia Test was associated with cortical volume in right medial orbitofrontal cortex (green), right lateral orbitofrontal cortex (green), right superior temporal cortex (blue), right middle temporal cortex (orange), and right temporal pole (light blue). (p < 0.01 corrected by Monte Carlo simulation).
