# Supplementary material for: Structural MRI study of Pareidolia and Visual Hallucinations in Drug–Naïve Parkinson’s disease
Source: Sci Rep. 2024 Dec 28;14:31293. doi: 10.1038/s41598-024-82707-x (PMC11682137; doi:10.1038/s41598-024-82707-x)
Supplement: Supplementary file 3 — Supplementary Information 3. [file 41598_2024_82707_MOESM3_ESM.pptx]

## Slide 1
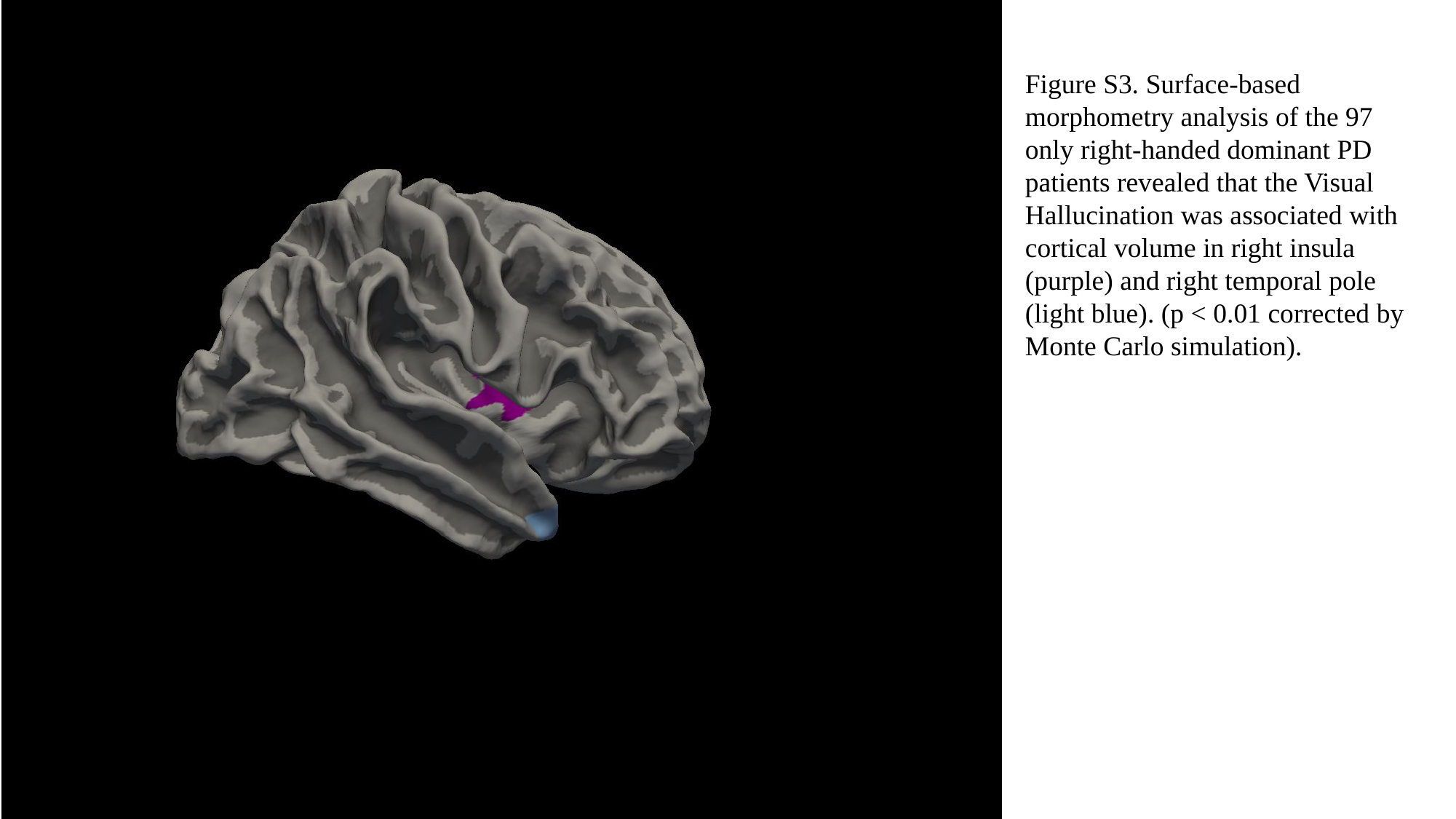

Figure S3. Surface-based morphometry analysis of the 97 only right-handed dominant PD patients revealed that the Visual Hallucination was associated with cortical volume in right insula (purple) and right temporal pole (light blue). (p < 0.01 corrected by Monte Carlo simulation).
