# Supplementary material for: Structural MRI study of Pareidolia and Visual Hallucinations in Drug–Naïve Parkinson’s disease
Source: Sci Rep. 2024 Dec 28;14:31293. doi: 10.1038/s41598-024-82707-x (PMC11682137; doi:10.1038/s41598-024-82707-x)
Supplement: Supplementary file 4 — Supplementary Information 4. [file 41598_2024_82707_MOESM4_ESM.pptx]

## Slide 1
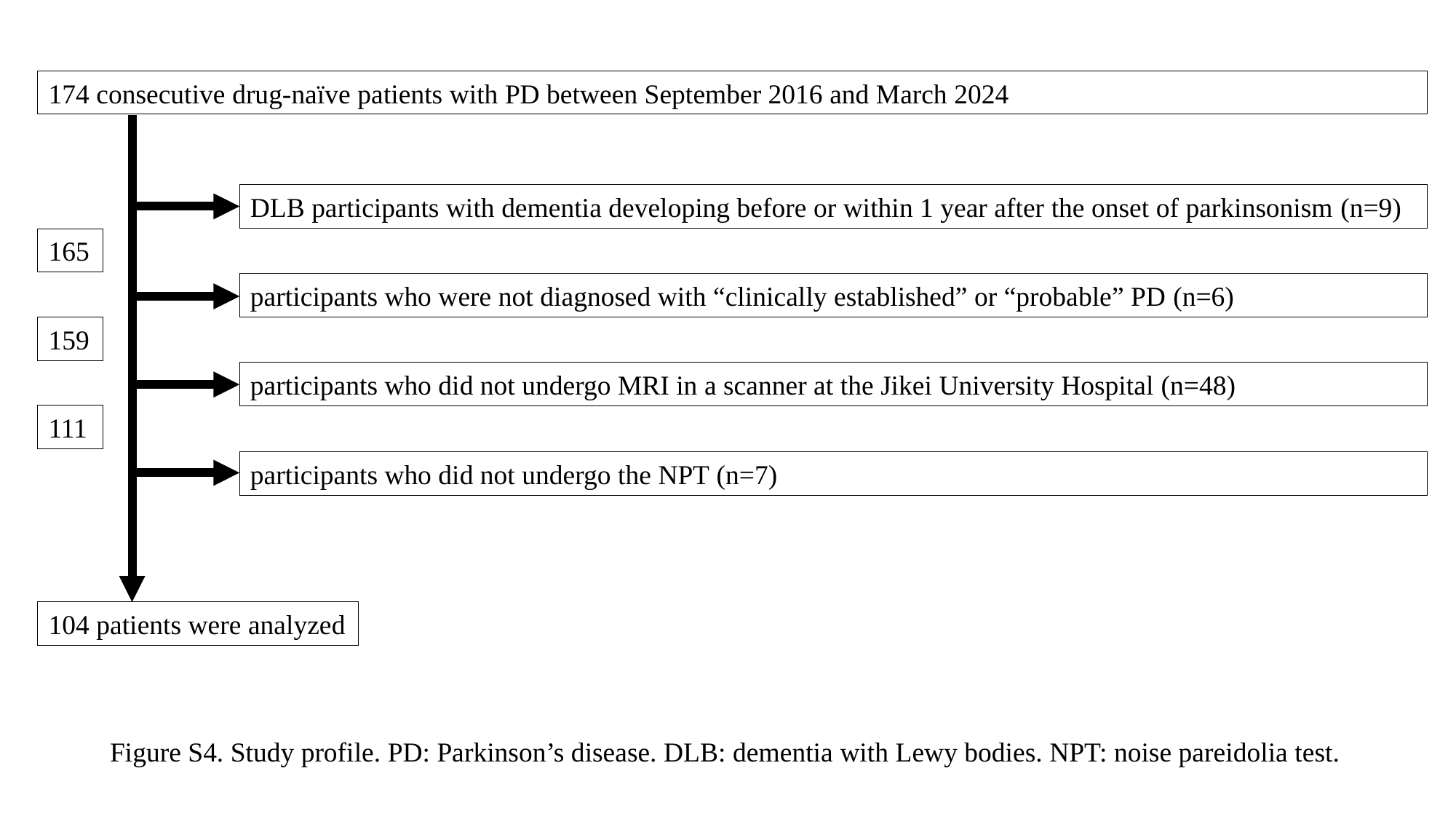

174 consecutive drug-naïve patients with PD between September 2016 and March 2024
DLB participants with dementia developing before or within 1 year after the onset of parkinsonism (n=9)
165
participants who were not diagnosed with “clinically established” or “probable” PD (n=6)
159
participants who did not undergo MRI in a scanner at the Jikei University Hospital (n=48)
111
participants who did not undergo the NPT (n=7)
104 patients were analyzed
Figure S4. Study profile. PD: Parkinson’s disease. DLB: dementia with Lewy bodies. NPT: noise pareidolia test.
